# The Oxidative Phosphorylation and Cytoskeleton Proteins of Mouse Ovaries after 96 Hours of Hindlimb Suspension

**DOI:** 10.3390/life13122332

**Published:** 2023-12-12

**Authors:** Elena Yu. Gorbacheva, Maria A. Sventitskaya, Nikolay S. Biryukov, Irina V. Ogneva

**Affiliations:** 1 Cell Biophysics Laboratory, State Scientific Center of the Russian Federation Institute of Biomedical Problems of the Russian Academy of Sciences, 76a, Khoroshevskoyoe shosse, Moscow 123007, Russia; elenagorbacheva22@gmail.com (E.Y.G.); biryukovns@gmail.com (N.S.B.); iogneva@yandex.ru (I.V.O.); 2 Gynecology Department, FGBU KB1 (Volynskaya) UDP RF, 10, Starovolynskaya Str., Moscow 121352, Russia; 3 Medical and Biological Physics Department, I. M. Sechenov First Moscow State Medical University, 8-2 Trubetskaya St., Moscow 119991, Russia

**Keywords:** OXPHOS, microgravity, ovary, cytoskeleton, gene expression

## Abstract

The purpose of this study was to assess oxidative phosphorylation (OXPHOS) in mouse ovaries, determine the relative content of proteins that form the respiratory chain complexes and the main structures of the cytoskeleton, and determine the mRNA of the corresponding genes after hindlimb suspension for 96 h. After hindlimb suspension, the maximum rate of oxygen uptake increased by 133% (*p* < 0.05) compared to the control due to the complex I of the respiratory chain. The content of mRNA of genes encoding the main components of the respiratory chain increased (cyt *c* by 78%, cox IV by 56%, ATPase by 69%, *p* < 0.05 compared with the control). The relative content of cytoskeletal proteins that can participate in the processes of transport and localization of mitochondria does not change, with the exception of an increase in the content of alpha-tubulin by 25% (*p* < 0.05) and its acetylated isoform (by 36%, *p* < 0.05); however, the mRNA content of these cytoskeletal genes did not differ from the control. The content of GDF9 mRNA does not change after hindlimb suspension. The data obtained show that short-term exposure to simulated weightlessness leads to intensification of metabolism in the ovaries.

## 1. Introduction

Exposure to space flight conditions affects all systems of the body, including the reproductive system. The condition of the organs of the reproductive system, most often, is not a limiting factor in space exploration; therefore, until now, the attention of researchers has not been focused on its study. At the same time, especially for the female body, the functioning of the reproductive system ensures not only the preservation of the species but also, to a large extent, determines healthy aging. The increasing proportion of women who have completed at least one space flight leads to the need to study the effects of weightlessness on the female reproductive system.

Data obtained after being in space flight conditions have a large scatter of parameters, which determines their unrepresentativeness [1,2]. To date, little data have been obtained from experimental studies on Earth, in bed-rest or dry immersion models. After 17 days of antiorthostatic bed rest, it was shown that the duration of the menstrual cycle did not change [3], but in a similar study, 3 out of 12 women had a decrease in progesterone concentration, indicating luteal phase deficiency [4]. After female subjects stayed for 5 days in “dry” immersion conditions, we also noted a decrease in basal progesterone and luteinizing hormone, but the main focus of the study was on the follicular phase and oocyte maturation. It was shown that after being in “dry” immersion, the content of anti-Mullerian hormone does not change, but inhibin B increases, which is produced by granulosa cells and can be a marker of an increase in their number. Moreover, the size of the antral follicles and the dominant follicle increases, which can be considered a positive criterion for a maturing oocyte [5], which requires direct experimental confirmation.

However, the data presented in the literature relate primarily to in vitro experiments. Cultivation of mouse oocytes for 6 h in a random positioning machine leads to reorganization of the cytoskeleton and redistribution of actin from the cortical layer to the center of the cell and tubulin from the center to the cortical cytoskeleton [6]. With longer (24 h) cultivation of human oocytes, it was shown by transmission electron microscopy that the perivitelline space and the thickness of the *zona pellucida* asymmetrically increase, resulting in the redistribution of cortical granules, the fusion of mitochondria, and a change in the morphology of the smooth endoplasmic reticulum [7].

Culturing encapsulated mouse follicles in simulated microgravity for 2 days did not affect follicle survival; in particular, follicle diameters increased in both groups, as did the proportion of antral follicles. However, the development of ultrastructural abnormalities was noted, which the authors believed caused chaotic granulosa cell polarity and a decrease in growth differentiation factor 9 (GDF9), which is secreted by oocytes, resulting in decreased oocyte quality in these follicles. Moreover, higher levels of reactive oxygen species were found in oocytes isolated from follicles that were cultured under simulated microgravity conditions [8].

With longer cultivation (for 4 days) of isolated preantral follicles, survival under simulated microgravity conditions decreased by more than 20% compared to the control. In addition, under microgravity conditions, the size of oocytes did not increase, although the size of the follicle increased in the same way as under 1 g conditions. However, in the same study, the authors showed that the number of follicles with developed layers of functionally active granulosa cells after simulated microgravity was significantly greater than in the control [9].

There are very few in vivo experiments aimed at studying the reproductive tissues of a mammal exposed to microgravity conditions in order to analyze the mechanisms of development of the observed changes. Hindlimb suspension of rats led to a decrease in the concentration of estradiol in the blood, while the content of pituitary hormones did not change, although its weight decreased [10]. After space flight (shuttle missions STS-131, 133, 135), according to Ronca A. et al., cycling stops, the *corpus lutea* is lost, and the expression of the estrogen receptor in the uterus decreases [11]. However, in a recent study of the mouse estrous cycle and ovarian gene expression aboard the International Space Station, it was shown that mice are in different phases of the estrous cycle (10 mice in the flight group: 6—estrus, 4—metestrus), which, according to the authors, indicates the preservation of cycling and, respectively, the fertility of these individuals [12].

Summarizing the above data from in vivo and in vitro experiments, we can conclude that exposure of mammals to microgravity conditions leads to insufficiency of the luteal phase and a decrease in progesterone levels, but the follicular phase has been practically not studied. A few results give reason to believe that the functional activity of ovarian tissue in terms of the supply of nutrients to the growing oocyte after the female body has been in conditions of simulated weightlessness can be increased. That is why we decided to test the oxidative phosphorylation (OXPHOS) of ovarian tissues of mice during the full estrous cycle under hindlimb suspension conditions in this study. The OXPHOS was considered a marker of functional activity, a decrease in which—along with disruption of mitochondrial structure—can lead to the initiation of apoptosis [13,14]. In addition, given that mitochondrial activity depends on the structure of the cytoskeleton [15,16,17,18], changes to which can lead to the formation of the structural and functional pattern of cells under microgravity conditions, we assessed the cytoskeletal protein content and its mRNA. Oocyte quality was assessed using GDF9 mRNA content as a marker [8].

## 2. Materials and Methods

### 2.1. Experimental Design

The experiment was carried out on the ovaries of 12-week-old BALB/c mice (n = 14). The effects of microgravity were reproduced using the standard Ilyin–Novikov hindlimb suspension model modified by Morey-Holton [19]. In this case, the animal was suspended by the tail so that the hind limbs did not touch the support, i.e., the body was at an angle of 30° relative to the horizontal plane, and the animal could move freely around the cage on its forelimbs. The animals received standard vivarium food and water ad libitum, the day/night regime was 12/12 h, and all animals were kept under standard conditions. Suspension was carried out for 96 h, which constitutes a complete estrous cycle.

Two groups were formed: control group C (n = 7, m = 27.9 ± 0.6 g) and hindlimb suspension group HS (n = 7, m = 27.1 ± 0.9 g).

The animals were euthanized using «Forran» inhalation anesthesia (Abbott, Alameda, CA, USA), and the ovaries were isolated and weighed. Then, one-half of the ovary was used to assess cellular respiration, and the other was immediately frozen in liquid nitrogen for subsequent protein and mRNA isolation.

### 2.2. Measuring Cellular Respiration by Polarography

For the cellular respiration assay, one-half of the mouse ovary from each experimental group was used, as described by Kuznetsov et al. [20]. Immediately after extirpation, the ovaries were digested and incubated with 10 μg/mL saponin (10 min at +25 °C with shaking). Then, the samples were added to a polarographic cuvette, and the change in the oxygen concentration was measured by an Oxygraph+ polarograph (Hansatech Instruments, Ltd., Norfolk, UK) at +25 °C.

To assess the contribution of each complex of the respiratory chain, inhibitors and substrates were added sequentially [20] with some modifications [21]. Briefly, at the start of the measure of each sample, we measured V_0_—the basal oxygen uptake rate. Then, we added NADH dehydrogenase substrates—10 mM glutamate and 5 mM malate—and recorded V_glu+mal_, after addition of 2 mM ADP—maximum respiration rate V_max_. After that, we sequentially added 0.5 μM rotenone (inhibits complex I), 10 mM succinate (the substrate of succinate dehydrogenase—the rate V_II_ was recorded), 5 μM antimycin A (inhibits cytochrome c reductase), 0.5 mM TMPD + 2 mM ascorbate (the artificial cytochrome c oxidase substrates—the rate V_IV_ was recorded). To avoid artifacts associated with damage to the outer mitochondrial membrane when samples were treated with saponin, its integrity was tested by adding 10 μM cytochrome. The rate of oxygen uptake was measured in pmol O_2_ per mL per min per mg of dry mass.

### 2.3. Estimation of the Relative Protein Content by Western Blotting

Total protein content was isolated from frozen ovaries. Tissues were cut into thin sections and then homogenized in Laemmli buffer with a protease inhibitor cocktail (Calbiochem, San Diego, CA, USA). SDS-electrophoresis was performed on polyacrylamide gels (Bio-Rad Laboratories, Hercules, CA, USA). After measuring the concentration of the total protein of each sample, equal amounts of protein were placed in each well, then electrophoresis was carried out to separate the proteins, and then they were transferred to a nitrocellulose membrane [22]. We used Ponceau staining as well as H3 for loading control. Specific primary monoclonal antibodies were used to determine the amount of each protein, according to Table 1.

Horseradish peroxidase-conjugated horse antibodies for chemiluminescent detection were used as secondary antibodies to detect mouse and rabbit IgG (Cell Signaling Technology, Danvers, MA, USA, #7076S and #7074S, respectively) at a dilution of 1:10,000, and Super Signal™ West Femto Maximum Sensitivity Substrate (Thermo Scientific, Waltham, MA, USA) was used for detection. The Fiji package (https://imagej.net/Fiji, accessed on 20 June 2023) was used to analyze protein bands.

### 2.4. Estimation of the Relative Content of mRNA by RT-PCR

RNeasy Micro Kit (Qiagen, Düsseldorf, Germany) was used for total mRNA isolation according to the instructions. For reverse transcription, 500 ng RNA and d(T)15 as a primer were used. Real-time PCR was performed using primers created by Primer-BLAST (https://www.ncbi.nlm.nih.gov/tools/primer-blast, accessed on 16 May 2023) (Table 2). The expression of target genes was normalized to histone H3 and quantified by the 2^−ΔΔCT^ method [23].

### 2.5. Statistical Analysis

Statistical processing of the data was carried out in ANOVA using a post hoc *t*-test with a significance level of *p* < 0.05 to assess the significance of the differences determined between groups. Data are presented as mean ± standard error of the mean (M ± SE). All the methods were carried out in accordance with the relevant guidelines and regulations.

## 3. Results

The weight of the ovaries after hindlimb suspension of mice during the full estrous cycle (96 h) did not change: 9.6 ± 0.8 mg in the control group and 9.0 ± 0.6 mg in the hindlimb suspension group.

### 3.1. OXPHOS of Mouse Ovaries, the Content of Proteins That Form Complexes of the Respiratory Chain, and the Content of mRNA of the Corresponding Genes

**Figure 1:**
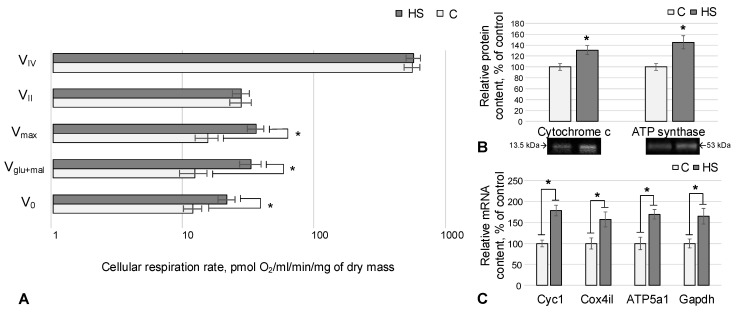
OXPHOS data and relative content of the respiratory chain proteins with corresponding mRNA content. C—control group, HS—hindlimb suspension group. *—*p* < 0.05 compared to the control group C. (**A**) Rate of cellular respiration in mouse ovaries. V_0_, V_glu+mal_, V_max_, V_II_, and V_IV_ are measured rates of oxygen uptake during substrate-inhibitor analysis (detailed description is in the Materials and Methods section). (**B**,**C**) Relative protein and mRNA content of the respiratory chain complexes: Cyc1—cytochrome c, Cox4il—cytochrome c oxidase, ATP5a1—subunit F1 of ATP synthase, Gapdh—glyceraldehyde-3-phosphate dehydrogenase.

After hindlimb suspension of female mice, the basal rate of oxygen uptake by permeabilized ovaries V_0_ increased by 81% (*p* < 0.05), the rate V_glu+mal_ (after the addition of substrates of the complex I)—by 169% (*p* < 0.05), in comparison with the control group (Figure 1A). With the addition of ADP, the maximum respiratory rate V_max_ also increased by 133% (*p* < 0.05) (Figure 1A). We did not observe statistically significant differences between the rates of oxygen uptake after inhibition of the NADH dehydrogenase complex and the addition of substrates of the second complex of the respiratory chain, as well as after inhibition of cytochrome c oxidase and the addition of complex IV substrates (Figure 1A).

The relative abundance of the electron transport protein, cytochrome c, increased after hindlimb suspension by 31% (*p* < 0.05) relative to the control group (Figure 1B). Also, in the HS group, there was an increase in the relative content of the catalytic subunit F(1) of ATP synthase by 45% (*p* < 0.05) compared with control group C (Figure 1B).

The mRNA content of genes encoding electron transport chain proteins, Cyc1, Cox4i1 and ATP5a1, increased by 78% (*p* < 0.05), 56% (*p* < 0.05), and 69% (*p* < 0.05), respectively, relative to control (Figure 1C). In addition, the amount of mRNA of the gene encoding the enzyme that catalyzes the reversible oxidative phosphorylation of glyceraldehyde-3-phosphate, Gapdh, increased by 65% (*p* < 0.05) relative to the control group (Figure 1C).

### 3.2. Relative Content of Cytoskeletal Proteins and mRNAs Encoding Their Genes

**Figure 2:**
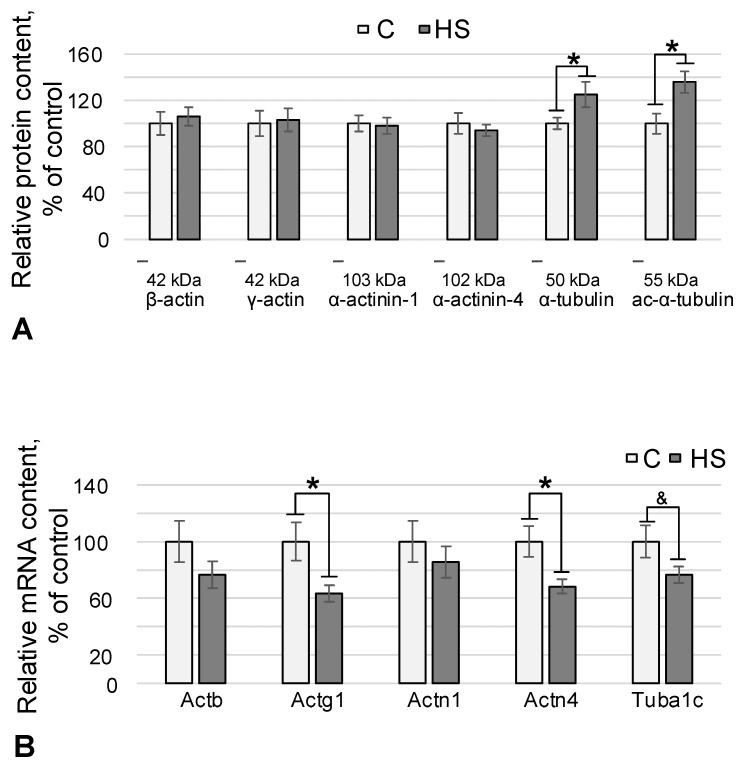
Relative content of cytoskeletal proteins and corresponding mRNAs in mouse ovarian tissues. C—control group, HS—antiorthostatic suspension group. *—*p* < 0.05 compared to the control group C, ^&^—*p* = 0.1 compared to the control group C. (**A**) Protein content—beta-actin (42 kDa), gamma-actin (42 kDa), alpha-actinin-1 (103 kDa), alpha-actinin-4 (102 kDa), alpha-tubulin (50 kDa), acetylated alpha-tubulin (55 kDa). (**B**) Relative mRNA content of genes encoding cytoskeletal proteins Actb, Actg1, Actn1, Actn4, Tuba1c—beta- and gamma-actin, alpha-actinin-1 and -4 and alpha-tubulin, respectively.

The relative content of actin isoforms (beta and gamma) and actin-binding proteins alpha-actinin-1 and alpha-actinin-4 did not change after hindlimb suspension relative to the control group (Figure 2A). At the same time, we observed an increase in the relative content of the acetylated isoform of alpha-tubulin by 36% (*p* < 0.05) after exposure to conditions simulating the effects of weightlessness (Figure 2A). The content of alpha-tubulin increased by 25% (*p* < 0.05) in the HS group relative to the control (Figure 2A).

The mRNA content of the Actb gene encoding beta-actin did not change against the background of a decrease in the mRNA content of the gene encoding gamma-actin by 37% (*p* < 0.05) in the HS group relative to the control (Figure 2B). The mRNA content of the genes encoding alpha-actinin-1 also remained unchanged, while the relative mRNA content of the gene encoding alpha-actinin-4 decreased by 32% (*p* < 0.05) in the hindlimb suspension group relative to the control group. The content of gene mRNAs encoding alpha-tubulin did not change significantly in the experimental group but had a tendency to decrease (*p* = 0.1) (Figure 2B).

### 3.3. Content of Gdf9 Gene mRNA in Mouse Ovaries after Hindlimb Suspension

**Figure 3:**
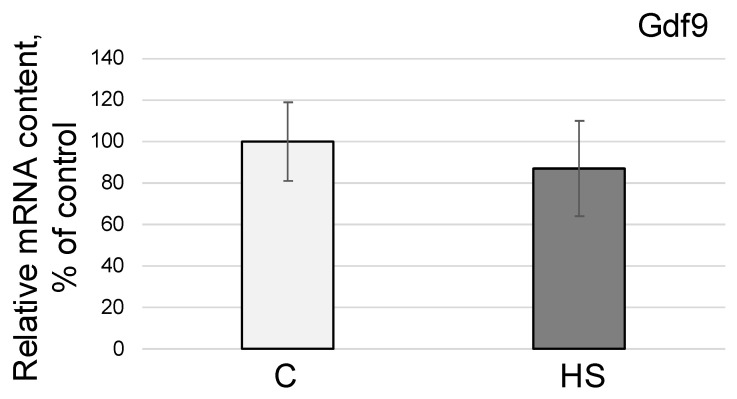
Relative mRNA content of the gene encoding GDF9. C—control group, HS—anti-orthostatic suspension group.

The mRNA of the gene encoding growth differentiation factor 9 (GDF9) did not change under conditions simulating the effects of weightlessness relative to the control group C (Figure 3).

## 4. Discussion

Research into the effect of weightlessness on the female reproductive system can, on the one hand, provide new approaches to protecting the female body during deep space exploration and maintaining health after returning to Earth, including from the age perspective. On the other hand, the possibility of using the results obtained in assisted reproductive technologies cannot be ruled out.

Our previously obtained data in a study of the hormonal status of female subjects after a 5-day stay in conditions simulating weightlessness (“dry” immersion) showed that the concentration of inhibin B in the blood and the dominant follicle’s diameter were increased in the next menstrual cycle [5], which may indicate an increase in the probability of a fully mature oocyte [24]. However, it is impossible to find out what the functional state of ovarian tissue is in experiments involving humans. In experiments on *Drosophila* exposed to simulated microgravity conditions for 79 h (corresponding to the oogenesis cycle in the fruit fly), we observed an increase in OXPHOS intensity, which is an indicator of the functional activity of the tissue [25]. But, the existing differences in the type of oocyte maturation and hormonal regulation do not allow us to extrapolate these results to mammals. Therefore, to assess the functional status of mammalian ovaries, mice were subjected to hindlimb suspension during the full estrous cycle (96 h).

The weight of animals and ovaries did not differ between animals in the control and experimental groups, but the rate of oxygen absorption by the ovaries of mice after hindlimb suspension significantly increased (Figure 1A). Inhibitory analysis suggests that the intensification of OXPHOS occurs due to complex I of the mitochondrial respiratory chain. The intensification of OXPHOS due to complex I indicates that mouse ovarian tissues under simulated microgravity avoid the Warburg effect, which, for example, appears in *Drosophila* ovaries under such conditions [25] and intensifies the usual type of energy supply.

It should be noted that complex I is the main source of reactive oxygen species in the cell [26]. Accordingly, increased oxidative phosphorylation, especially due to complex I, can lead to an increase in reactive oxygen species, which was shown in an in vitro experiment when encapsulated follicles were exposed to simulated microgravity conditions [8]. Moreover, it should be noted that an increase in oxidative stress is associated with a decrease in the expression of proteins that form complexes I, III, and IV of the mitochondrial respiratory chain in the rat hippocampus under antiorthostatic suspension [27]. However, on the other hand, the development of oxidative stress occurs when there is an imbalance in the production of ROS and the antioxidant system [28] and, moreover, can lead to abnormal mitochondrial fission [29]. According to electron microscopy data, when cells were exposed to simulated microgravity, the fissions took place, but the number of mitochondria did not change [7]. This can be achieved by increasing the content of other respiratory chain complexes.

To test this assumption, we determined the content of key components of the respiratory chain—cytochrome c and ATP synthase—and observed their increase (Figure 1B), probably due to an increase in the mRNA content of the corresponding genes (Figure 1C). A similar strategy for increasing the intensity of OXPHOS by increasing the copy number of mRNA of genes encoding respiratory chain complexes is also typical for other tissues of mice, in particular the heart [30,31].

However, a question remains regarding the mechanism by which the mRNA content of mitochondrial proteins increases under microgravity conditions. The regulation of the structural and functional pattern of mitochondria in different species and in different tissues is often determined by their interaction with the cytoskeleton [16,17,18]. For example, in cultured human lymphocyte cells (Jurkat) under real weightlessness conditions, a change in mitochondrial localization occurred due to degradation of cytoskeletal proteins, and changes in cristae morphology were observed, which may indicate impairment of mitochondrial function [32].

Therefore, we determined the content of key cytoskeletal proteins. Actin is involved in mitochondrial localization [33], but its content in mouse ovarian tissue did not change after hindlimb suspension (Figure 2A), and neither did the content of actin-binding proteins that organize microfilaments into a network—alpha-actinin-1 and alpha-actinin-4. Microtubules formed by tubulin are involved in the transport of mitochondria and the establishment of their localization [16,34]. After hindlimb suspension, the content of alpha-tubulin (Figure 2A) and its acetylated isoform (a marker of stable microtubules) increased, which correlates with the observed increase in OXPHOS intensity.

Similar to the increase in the content of proteins that form complexes of the respiratory chain, we assumed that an increase in the content of alpha-tubulin could be associated with an increase in the content of mRNA. Surprisingly, Tuba1c mRNA in mouse ovarian tissues after 96 h of hindlimb suspension did not differ significantly from the control but had a slight tendency to decrease (Figure 2B). In addition, when the content of actin and actin-binding proteins remains unchanged, the content of gamma-actin and alpha-actinin-4 mRNA decreases (Figure 2B).

This protein-mRNA pattern may indicate regulation of the content of cytoskeletal proteins at the level of translation/proteolysis. At the same time, hindlimb suspension for a longer duration—23 days—leads to a change in this balance: against the background of a constant content of cytoskeletal proteins, the content of their mRNA becomes higher than in the control [35]. We can assume that such dynamics of changes in mRNA content may be associated with epigenetic events, in particular, with the establishment of hypomethylated DNA status in the mouse ovaries after 23 days of hindlimb suspension [35]. This is a speculative assumption, although it has been shown that during space flight, the level of DNA methylation in preimplantation embryos decreases [36]. Moreover, the dynamics of changes in 5hmC and a decrease in the level of methylation of certain regions of DNA in human lymphoblastoid cells have been shown [37], possibly as a result of an increase in the activity of demethylases, which was shown in lymphocytes under simulated micro-gravity [38].

Considering the expression of GDF9, which is produced by the oocyte and regulates the growth of granulosa cells [39], as a marker of the state of the growing oocyte, we found no differences between the group after 96 h hindlimb suspension and the control group (Figure 3). However, data from in vitro experiments under simulated microgravity conditions indicate a decrease in GDF9 expression, which is associated with structural changes in the oocyte and its environment [8]. Moreover, culturing mouse oocytes for even 6 h under simulated microgravity conditions leads to reorganization of the cytoskeleton and changes in gene expression [6]. However, it is possible that under in vivo conditions, the role of factors leading to an increase in the number of granulosa cells, which provide nutrition to the growing oocyte, is to help maintain its quality. Moreover, in a real space flight in vivo study of mice, it was shown that the expression of GDF9 in mouse ovaries did not change relative to the control [12].

## 5. Conclusions

Research into the cellular mechanisms and pathogenesis of the reaction of the female reproductive system to being in conditions of weightlessness is in the phase of accumulating experimental data. Results from in vivo studies in mammals are extremely limited. Our results give reason to cautiously assume that exposure of female mice to hindlimb suspension conditions during the estrous cycle leads to intensification of OXPHOS in the ovaries against the background of an increase in the content of mRNA of genes encoding the main components of the mitochondrial respiratory chain. In this case, the content of cytoskeletal proteins that can influence the localization of mitochondria does not change; however, their transportation may be more efficient due to an increase in the content of tubulin and its acetylated isoform. At the same time, the maintenance of such a cytoskeletal pattern is apparently due to regulation at the level of translation/proteolysis since the mRNA content of these cytoskeletal genes remains unchanged. However, the expression of GDF9, which can be considered a marker of oocyte quality, does not change after hindlimb suspension.

Summarizing the results obtained, we can assume that short-term exposure of mice to conditions of simulated weightlessness leads, rather, to increased metabolism of ovarian tissue against the background of an unchanged structural pattern, but, of course, requires further comprehensive study.

## 6. Limitations of the Study

In vivo studies of the effects of microgravity on the female reproductive system of rodents are limited to the antiorthostatic suspension model, which was primarily developed for the study of skeletal and muscular systems. The use of this model for the reproductive system is possible [10,40], in particular, as a less stressful effect than space flight [41]. However, additional validation of structural and functional changes in the reproductive system of females during suspension and in real space flight is required.

The main purpose of the study was to determine the rate of oxygen absorption by ovarian tissue immediately after antiorthostatic suspension. Ovarian tissue consists of many types of cells, and to identify the contribution of each of them to the observed increase in the rate of cellular respiration, it was necessary to conduct separate measurements of at least the cortical and medulla layers. However, this will significantly delay the dissection procedure and may affect the results of polarography. In addition, changes in protein content should be correlated with different cell types by immunohistochemical staining. For such a study, it is necessary to use at least one whole ovary, which will then be unsuitable for other studies. Since this work focused on cellular respiration, such research should be carried out in the future.

Finally, we did not determine the phase of the estrous cycle using histological staining. The suspension duration was only 96 h, which is unlikely to cause significant desynchronization. In addition, women undertake space flights, both long and short, which can coincide with different phases of the cycle if they do not stop it. On the other hand, data from a 37-day space flight show that cycling in mice continues, and the expression of the important enzyme aromatase depends on the phase of the cycle [12]. Therefore, the lack of a histologically confirmed cycle phase is a limitation of this study.

## Figures and Tables

**Table 1:** Primary antibodies.

Primary Antibodies	Molecular Weight	Dilution	Producer	Catalog Number
Cytochrome c-1	13.5 kDa	5 µg/mL	Abcam, Cambridge, UK	#ab13575
ATP synthase F1	53 kDa	1 µg/mL	Abcam, Cambridge, UK	#ab14748
Beta-actin	42 kDa	1:500	Santa Cruz Biotechnology, Inc., Dallas, TX, USA	#sc-81178
Gamma-actin	42 kDa	1:500	Santa Cruz Biotechnology, Inc., USA	#sc-65638
Alpha-actinin-1	103 kDa	1:500	Santa Cruz Biotechnology, Inc., USA	#sc-17829
Alpha-actinin-4	102 kDa	1:500	Santa Cruz Biotechnology, Inc., USA	#sc-393495
Alpha-tubulin	50 kDa	1:1000	Abcam, Cambridge, UK	#ab52866
AcetylatedAlpha-tubulin	55 kDa	1:500	Santa Cruz Biotechnology, Inc., USA	#sc-23950
Histone H3	15 kDa	1 µg/mL	Abcam, Cambridge, UK	#ab10799

**Table 2:** Primer sequence and product size.

Gene	Primer Sequence, Forward/Reverse (5′…3′)	Product Size, bp
Cyc1	GTGGAACCCTGGAACCCATA/CAAACAGTGCTGCCAGGTTTT	106
Cox4i1	CTTCCCTGATTCCCGCGATG/ACACTCCCATGTGCTCGAAG	208
ATP5a1	GGCAACCACAAGGTCGATTC/CGGACGACTGGCACAAAATG	241
Gapdh	TCCCAGCTTAGGTTCATCAGG/ATGAAGGGGTCGTTGATGGC	165
Actb	TGAGCTGCGTTTTACACCCT/TTTGGGGGATGTTTGCTCCA	231
Actg1	CTGGTGGATCTCTGTGAGCA/TCAGGAGGGAAGAAACCAGA	184
Actn1	AAACCTGAACACGGCCTTTG/ATTGACCGCCAACACTTTGC	199
Actn4	AATCCAATGAGCACCTTCGC/TGGTGTGCTTGTTGTCGAAG	243
Tuba1c	GGCTCGCCTAGATCACAAGT/CTCATCGTCTCCTTCAGCACT	172
Gdf9	CCTCTACAATACCGTCCGGC/CTGTAAAGGCCTCCAGGTGG	511
H3f3a	CCTCGGTGTCAGCCATCTTT/GCCATGGTAAGGACACCTCC	140

## Data Availability

All data generated or analyzed during this study are included in this article.
